# Supplementary material for: Knocking Out Rap1a Attenuates Cardiac Remodeling and Fibrosis in a Male Murine Model of Angiotensin II-Induced Hypertension
Source: Cells. 2025 Nov 20;14(22):1834. doi: 10.3390/cells14221834 (PMC12651711; doi:10.3390/cells14221834)

Supplement Figure S1. Representative M-mode Echocardiography Images WT Saline Day 0

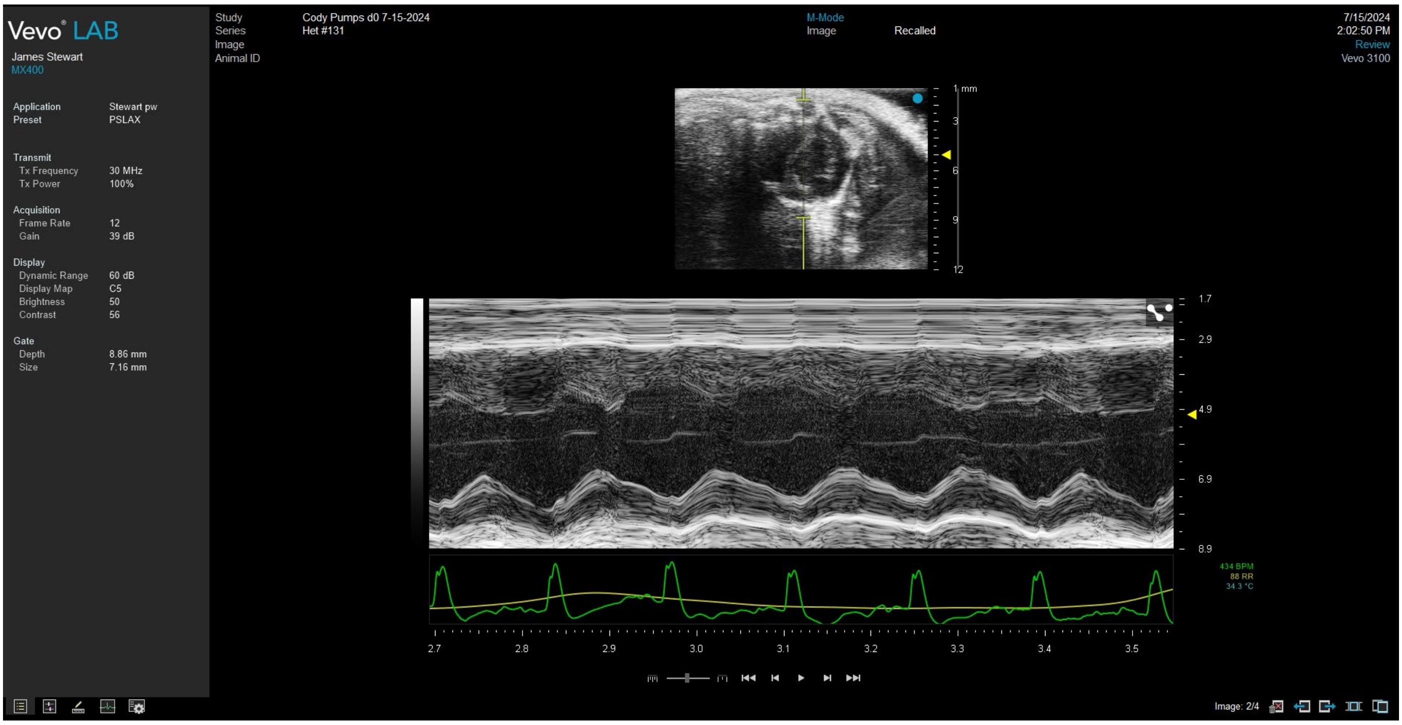

WT Saline Day 14

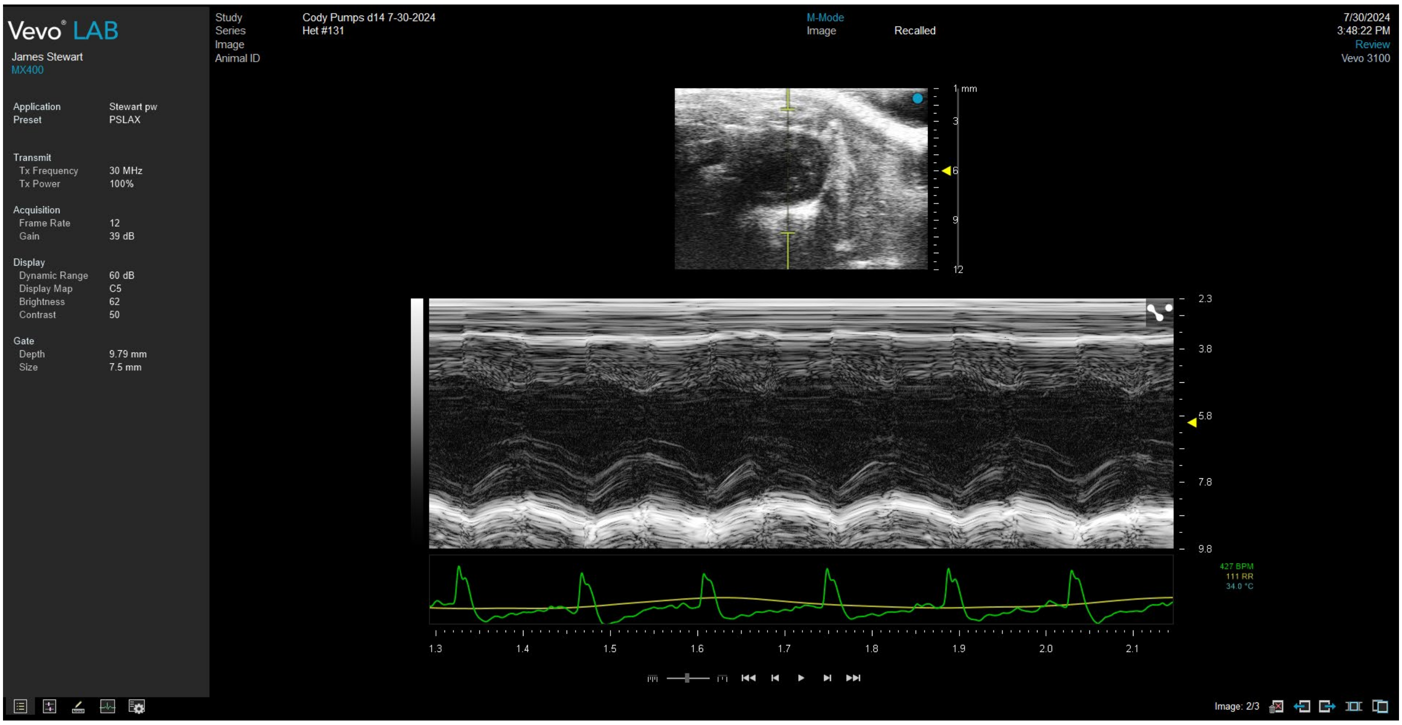

Supplement Figure S1. Representative M-mode Echocardiography Images WT AngII Day 0

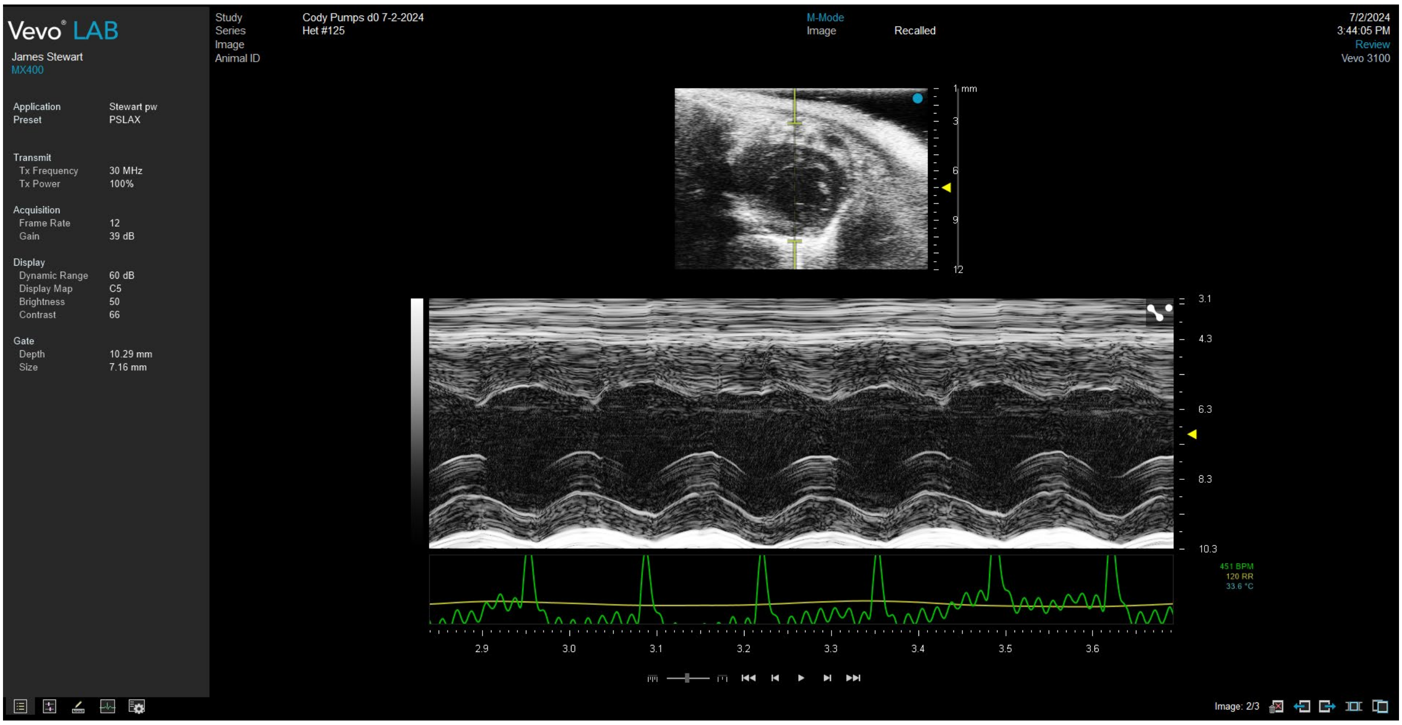

WT AngII Day 14

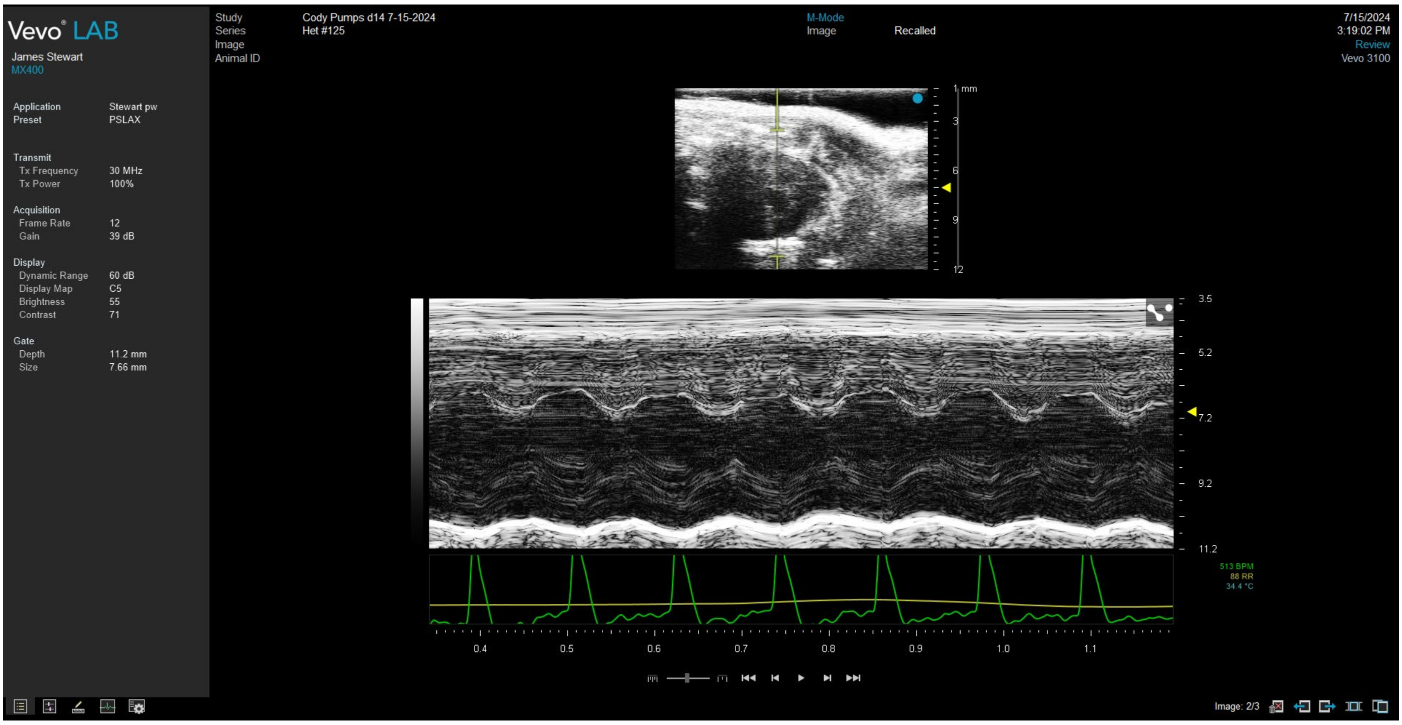

Supplement Figure S1. Representative M-mode Echocardiography Images RAGE KO Saline Day 0

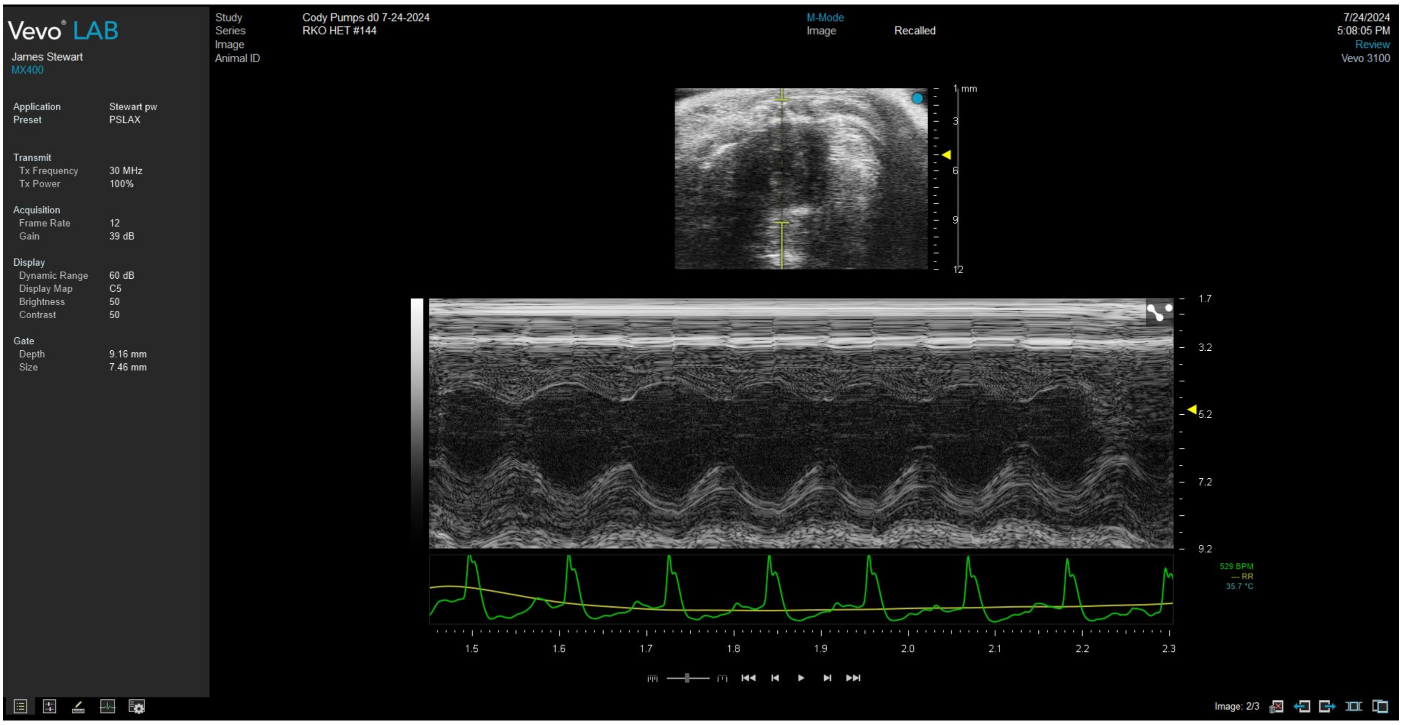

RAGE KO Saline Day 14

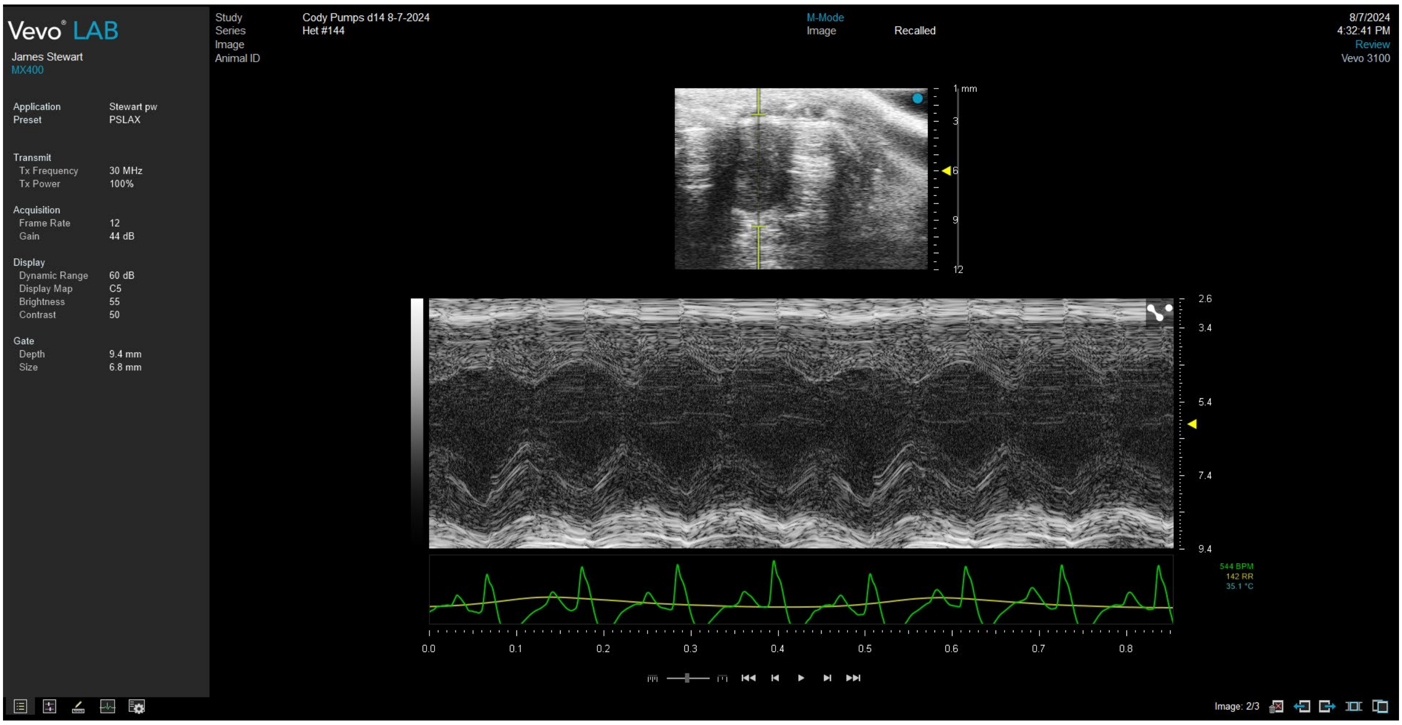

Supplement Figure S1. Representative M-mode Echocardiography Images RAGE KO AngII Day 0

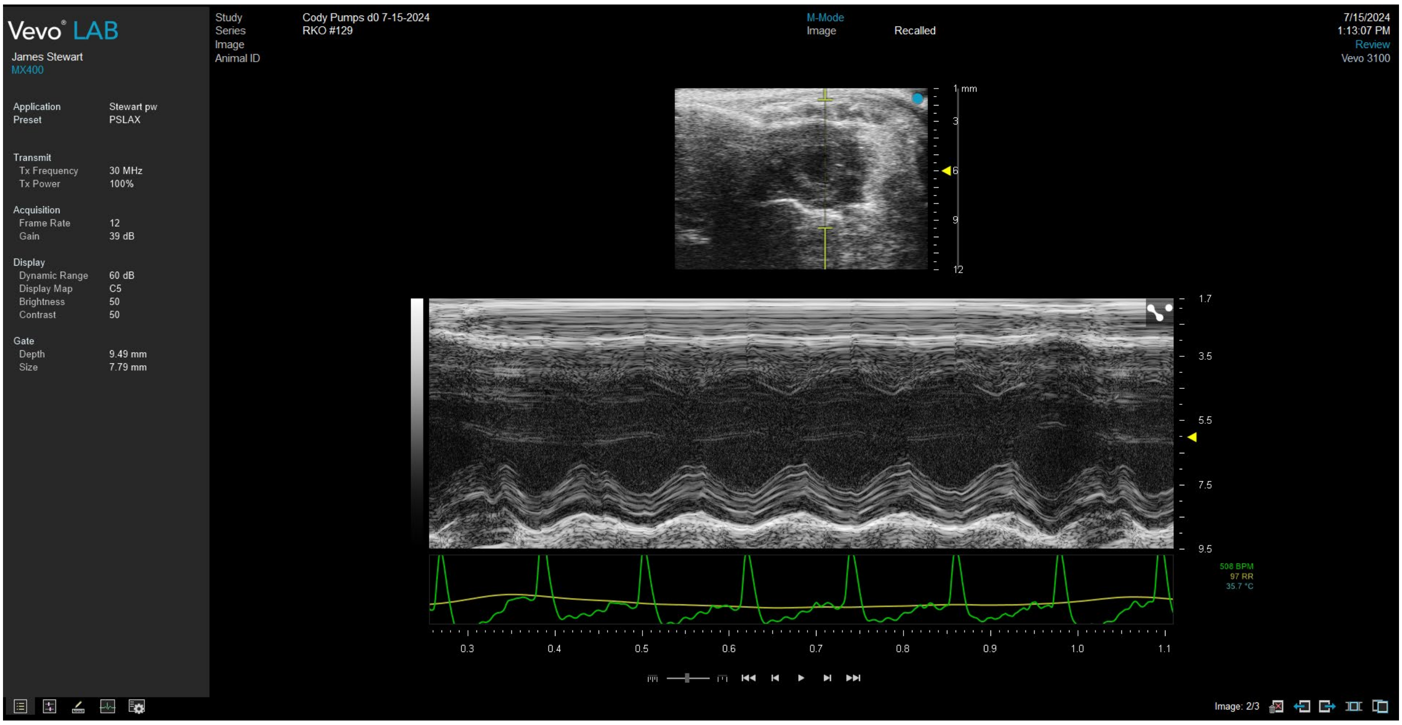

RAGE KO AngII Day 14

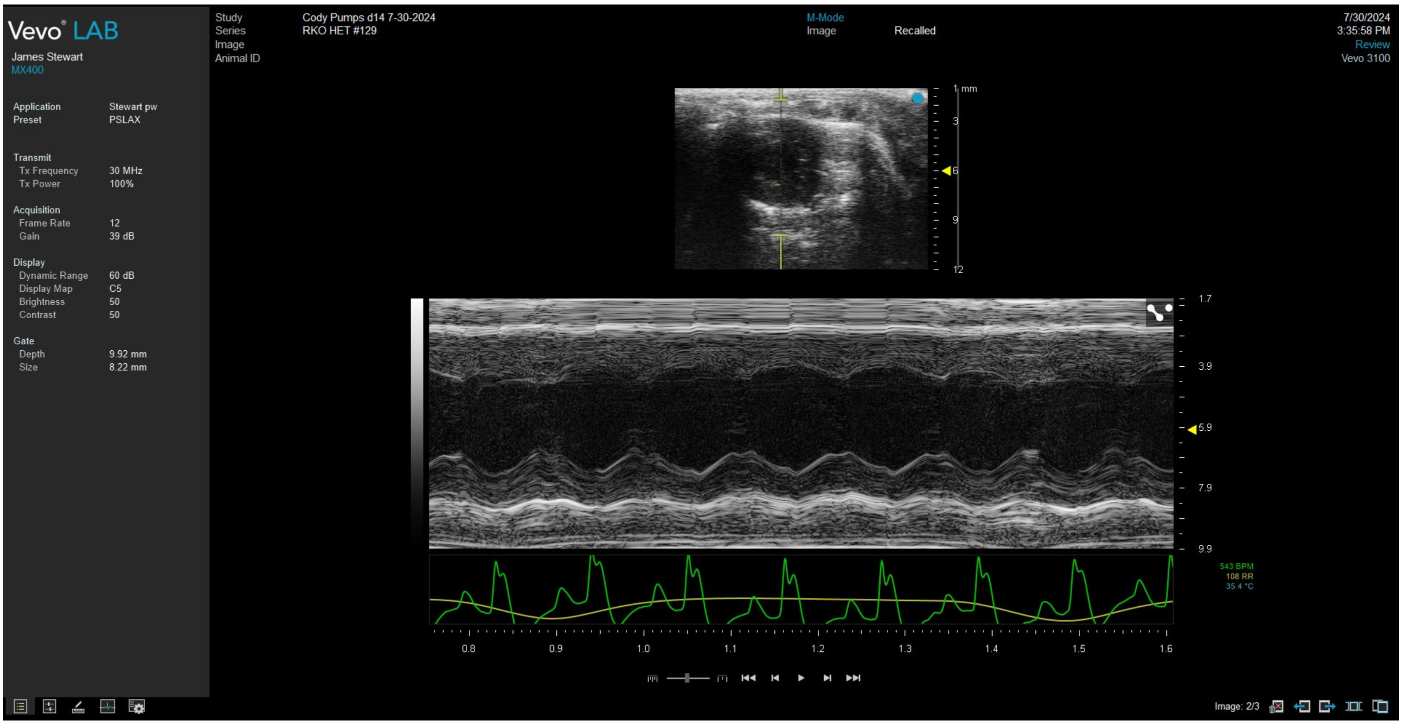

Supplement Figure S1. Representative M-mode Echocardiography Images RapKO Saline Day 0

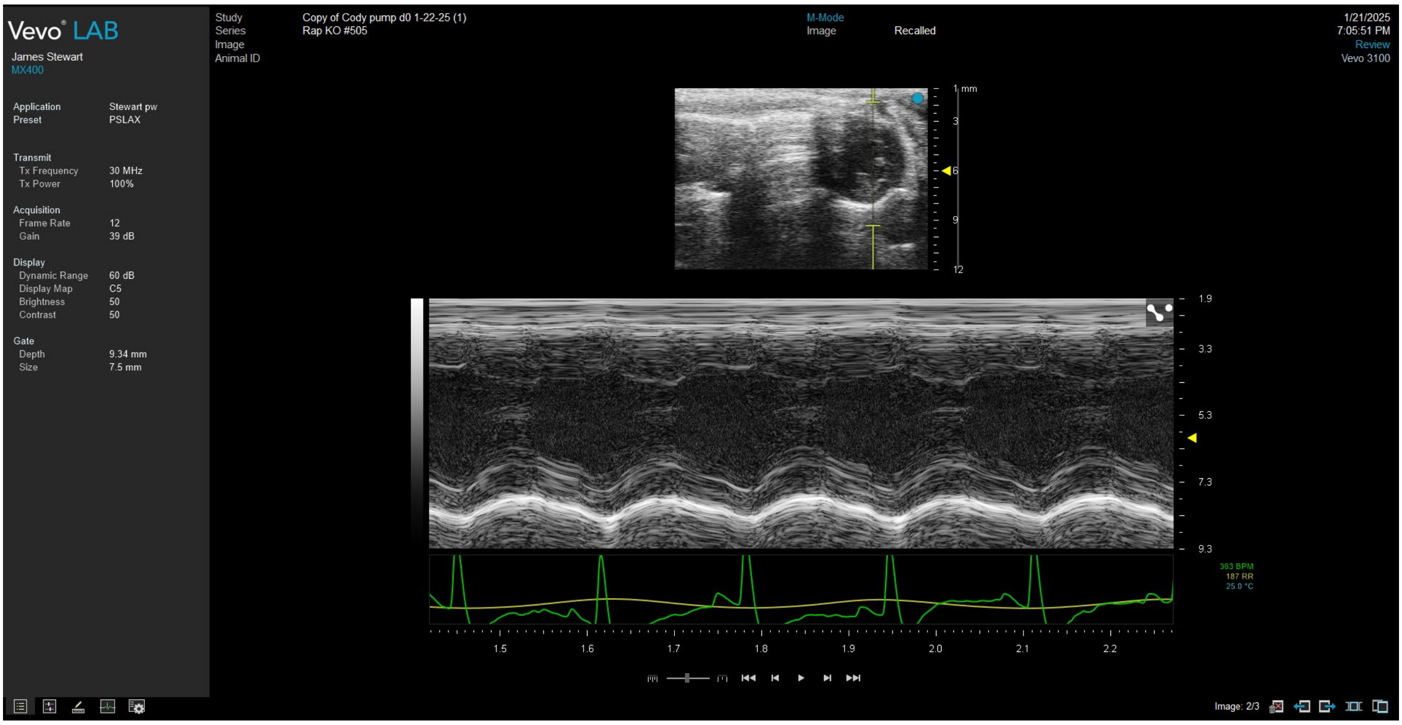

RapKO Saline Day 14

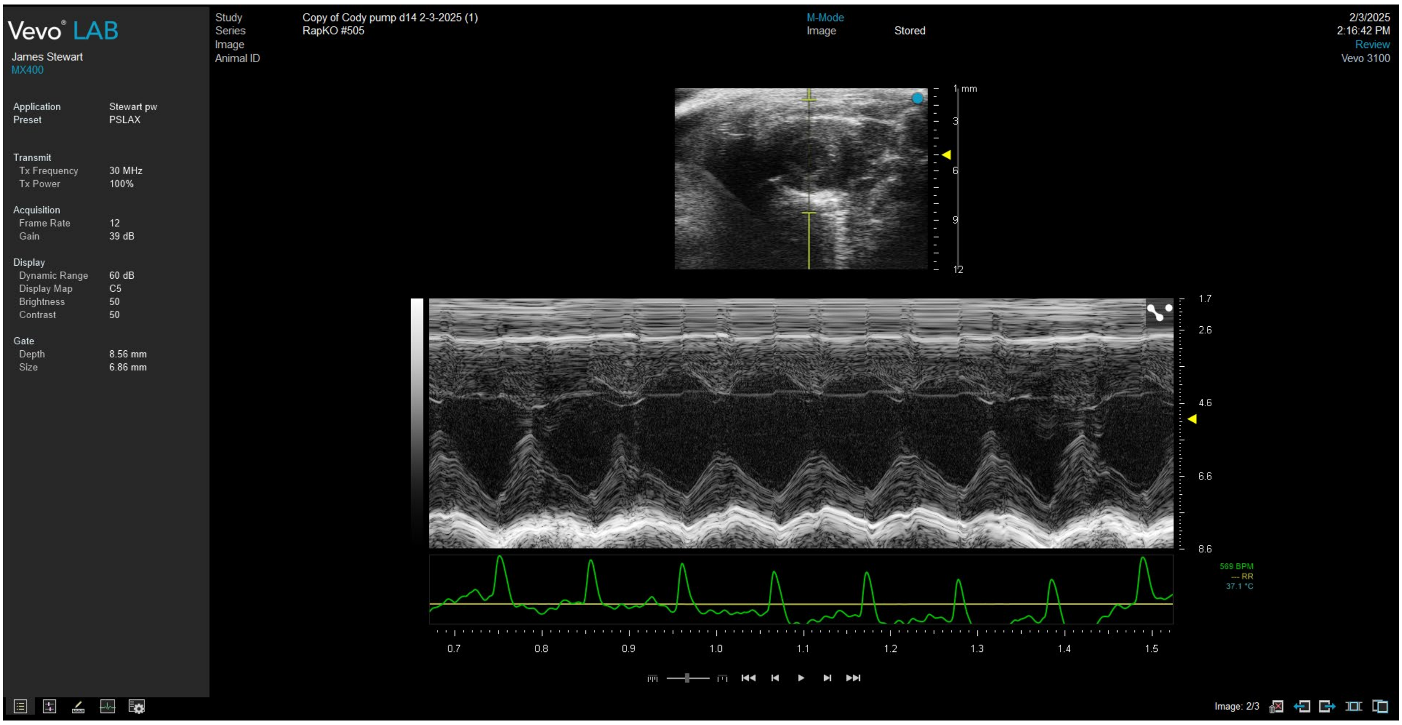

Supplement Figure S1. Representative M-mode Echocardiography Images RapKO AngII Day 0

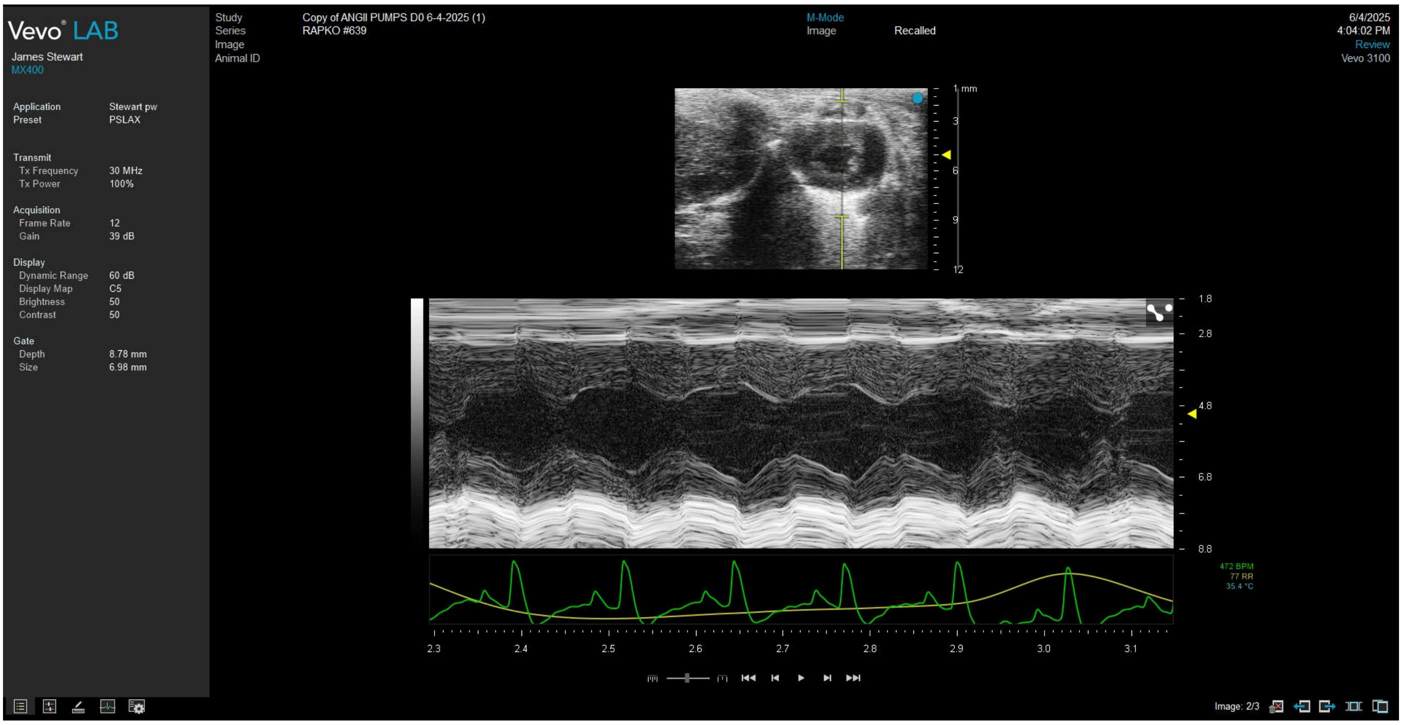

RapKO AngII Day 14

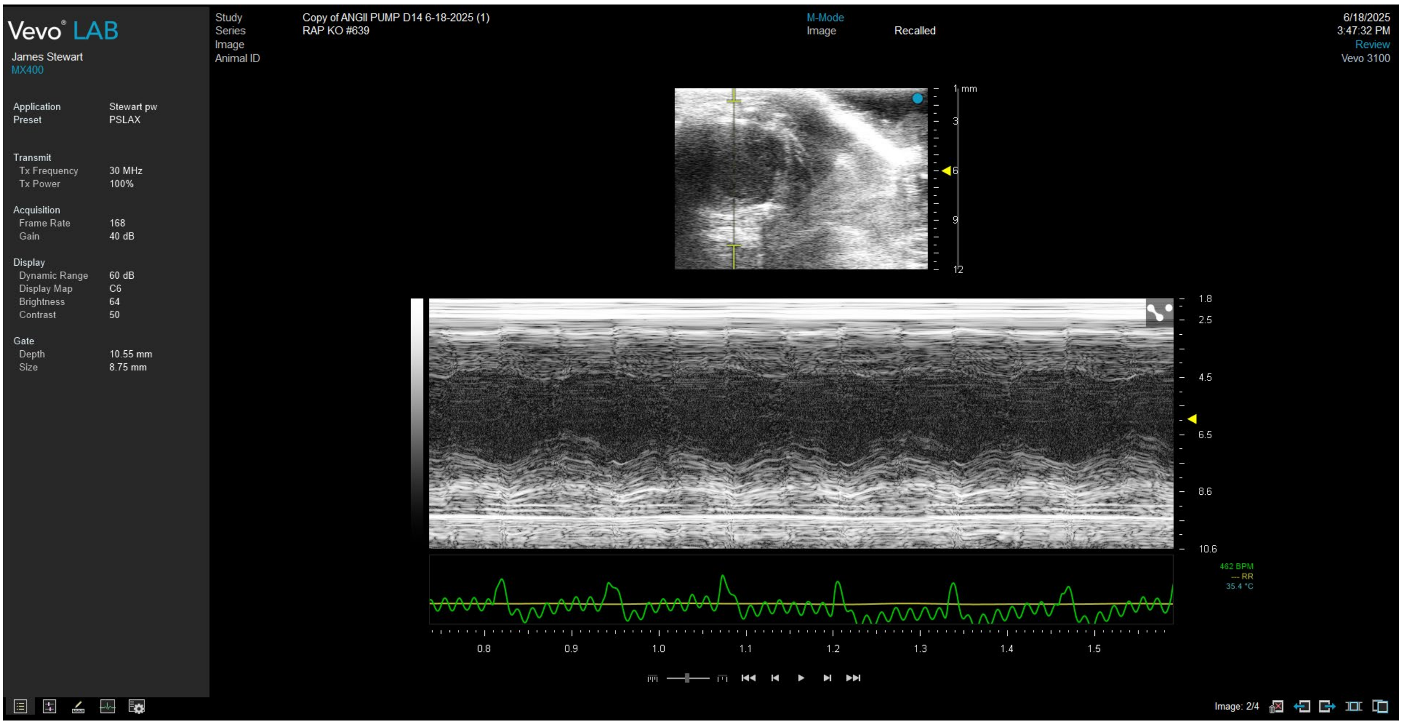

Supplement Figure S2. Representative Histology Images

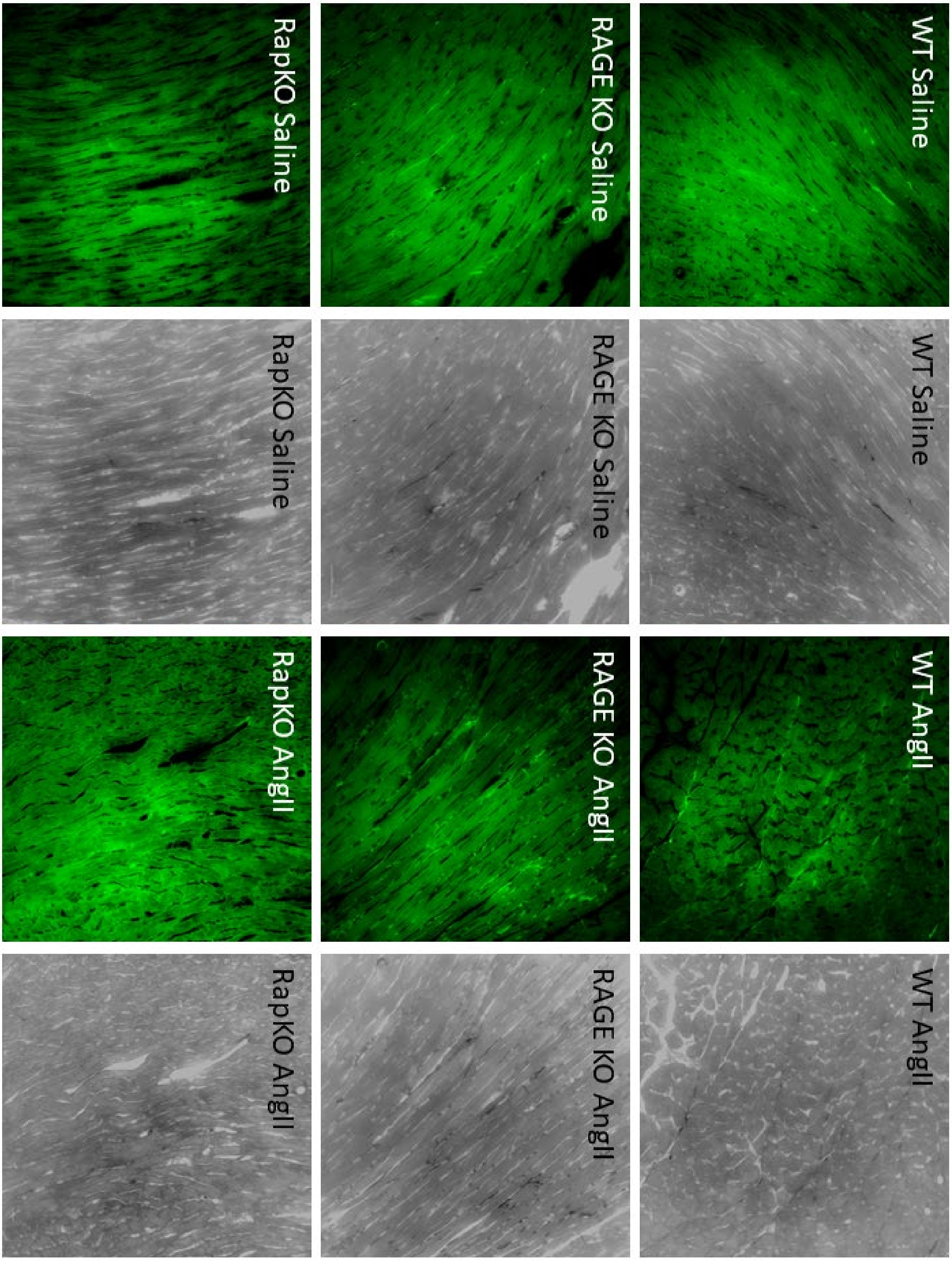

Supplement: Supplementary file 1 [file cells-14-01834-s001.zip › cells-3987998-supplementary.pdf]
